# Assessment of CVD- and PVD-Coated Carbides and PVD-Coated Cermet Inserts in the Optimization of Surface Roughness in Turning of AISI 1045 Steel

**DOI:** 10.3390/ma13225231

**Published:** 2020-11-19

**Authors:** Evandro Paese, Martin Geier, Fabiano R. Rodrigues, Tadeusz Mikolajczyk, Mozammel Mia

**Affiliations:** 1 Departamento de Engenharia Mecânica, Universidade de Caxias Sul, Campus Universitário da Região dos Vinhedos, Bento Gonçalves, Rio Grande do Sul 95705-266, Brazil; epaese@ucs.br; 2 Departamento de Engenharia Mecânica, Universidade Federal do Rio Grande do Sul, Porto Alegre, Rio Grande do Sul 90050-170, Brazil; martin.geier@ufrgs.br; 3 Programa de Pós-Graduação em Engenharia Mecânica, Universidade de Caxias Sul, Caxias do Sul, Rio Grande do Sul 95070-560, Brazil; frrodri1@ucs.br; 4 Department of Production Engineering, UTP University of Science and Technology, 85-796 Bydgoszcz, Poland; tami@utp.edu.pl; 5 Department of Mechanical Engineering, Imperial College London, South Kensington, London SW7 2AZ, UK

**Keywords:** surface roughness, coated carbide inserts, cermet inserts, turning mild steel, ANOVA, RSM, high material removal rate

## Abstract

In this study, an experimental and statistic investigation approach based on analysis of variance (ANOVA) and response surface methodology (RSM) techniques was performed to find the significant main effects and two-factor interaction effects and to determine how the controllable factors such as cutting speed, feed rate, depth of cut (DOC), tool nose radius, substrate and coating method of cutting tools influence surface quality in turning of AISI 1045 steel. The first optimal or near-optimal conditions for the quality of the generated surface and the second ones, including maximum material removal rate, were established using the proposed regression equations. The group mean roughness of the turned workpieces was lower from using chemical vapor deposition (CVD)-coated carbide inserts than the group means of other types of inserts; however they could not achieve the specific lowest roughness. The physical vapor deposition (PVD)-coated carbide and cermet inserts achieved the best surface quality when the specific combinations within the range interval of controllable factors were used in the experiment, showing that they may be applied to finish turning processes or even to particular high material removal rate conditions associated with the lowest roughness.

## 1. Introduction

The presence of irregularities in the generated surface produced in turning processes has numerous influences on several engineering applications. The surface finish of the machined parts is known to have a significant influence on wear, production costs and fatigue strength [1,2]. Few studies have analyzed the influence of cutting insert substrates and coating methods along with geometry and cutting parameters on the productivity of machining processes regarding surface finish [3,4]. Aiming to minimize production costs, the process’s controllable factors must be defined in a way that minimizes the production time and meets the project’s specifications, such as those of surface roughness [5,6]. Although cutting parameters’ influence in turning processes are well understood, the development of new cutting tool geometry, tool substrates and coating methods requires a continuous reassessment of its role in the process performance [7,8].

A comparative study on the effect of cutting conditions on machinability of Inconel 718 during high-speed turning with coated and uncoated polycrystalline cubic boron nitride (PCBN) tools was carried out by Bushlya et al. [9], where it was observed that the coated tools generated higher values of surface roughness. This behavior was attributed to variations in the tools’ microgeometry, where 3D optical measurements verified that the coated tools presented a greater cutting edge radius, which, in association with the other selected cutting parameters, affected the chip formation mechanism and, consequently, the machined surface roughness. A more in-depth analysis of surface quality, including surface roughness, concomitant with a metallographic analysis of the surface layer and induced plastic deformation during the turning of Inconel 718 was proposed by Zhou et al. [10]. Higher values of surface roughness were obtained with a coated cubic boron nitride (CBN) cutting tool compared to an uncoated one, and the main reason for this was attributed to the largest cutting edge radius, 20 µm and 15 µm respectively, showing that greater amounts of built-up edge on the machined surface occurred when the coated CBN cutting tool was used, mainly with lower feed rates. In [11], the increased cutting edge radius due to the presence of several coating layers on a tool coated by using a multilayer chemical vapor deposition (CVD) method was also attributed to a higher roughness of the machined surface of Incoloy 825 compared to uncoated tools.

An influence study of uncoated, and physical vapor deposition (PVD)- and CVD-coated tools on surface roughness in machining of Incoloy 825 was presented in [12], which verified that the surface roughness was higher when machined with the CVD-coated tool and lowest for the PVD-coated tool. The CVD coating process results in a rougher tool surface and an increased cutting-edge radius, which contributed to the lower surface finishing of the machined part. The high surface roughness of the part was also attributed to the greater friction in the tool–part interface. A better surface finish was achieved by tools coated with the PVD method, which was attributed to thinner coating and superior tribological properties.

The experimental approach of investigating one variable at a time has been replaced by mathematical modeling techniques to assist in the determination of optimal or close-to-optimal cutting conditions regarding the most varied and desired criteria, such as surface roughness, cutting force, wear of tool and others. Techniques such as Factorial Design, ANOVA, RMS, Taguchi and Artificial Neural Network have been widely used to reduce the cost and time of experimental analyses [4,13,14,15]. Mathematical models for assessing surface roughness versus cutting parameters of inserts with the same substrate but different coating methods (CVC/PVD) in turning operations of AISI P20 steel were proposed by Cakir et al. [16], where, although experiments showed a positive effect on surface roughness for higher cutting speeds when using CVD coating, the best surface finish was achieved when using PVD coating. Khellaf et al. [17] presented a comparative analysis of uncoated and coated ceramic cutting tools in hard turning of AISI H11 steel in order to optimize machined surface quality. A proposal for optimizing surface finishing was obtained using RSM and ANOVA methods, showing that the uncoated ceramic tool performed better in the considered cutting conditions. The application of CBN-coated cutting tools, known commercially as CBN7020 grade, was analyzed in hard turning of AISI 52100-bearing steel by Bouacha et al. [18]. This study included an evaluation of the surface roughness and cutting force in relation to the cutting parameters using RMS and ANOVA techniques. The main and interaction effects of controllable factors (cutting speed, feed rate and depth of cut) on response variables were evaluated, showing that the surface roughness is highly affected by the feed rate, whereas the cutting speed has a negative effect and the depth of cut demonstrates a negligible influence. Toulfatzis et al. [19] designed experiments with the Taguchi method to reduce the number of experiments and subsequently applied ANOVA for optimizing the surface quality in turning lead-free brass alloys using an uncoated carbide tool. The work verified the significance of four controllable factors (cutting speed, depth of cut, feed rate and workpiece material), each at four levels. When classifying the obtained contributions, the sequence of the four factors that affected the surface roughness in decreasing order was as follows: feed rate, depth of cut, type of workpiece material and cutting speed. Effects of ceramic and CBN cutting tools and cutting parameters on surface roughness were investigated by [20] in 718 nickel superalloy by applying a two-level full factorial design of experiment with a subsequent analysis of factor effects using an algorithm based on extended design matrix. The results showed an advantage for the ceramic insert in the surface roughness when compared to the CBN ones for both high and low feed rates considered in the study.

Kuntoglu et al. [21], performed a multi-criteria optimization of surface roughness and vibration based on an RMS approach. The optimum process parameters were predicted and experimentally verified to obtain the lowest roughness. The axial vibration showed similar behavior to surface roughness. The feed rate demonstrated a dominant influence on both feed rate and axial vibration, validating a similar variation. Moreover, Kuntoglu et al. [22], presented a multi-optimization and analysis of surface roughness, flank wear and different data sensors to a lathe, including dynamometer, vibration, acoustic emission, temperature and motor current sensors. Based on an RMS optimization approach, the optimum cutting conditions were predicted and experimentally verified to obtain the best results for flank wear, surface roughness and the sensorial data, with an 82.5% success rate for total desirability.

An optimization design of cutting parameters when turning Inconel 718 with cermet inserts was performed in [23]. The analysis consisted of applying the RSM, which used the analysis of variance (ANOVA) method, to develop a mathematical model for surface roughness based on cutting parameters. It was found that the cutting speed presents the strongest effect on the surface roughness among the selected parameters and is inversely proportional to the response. A similar methodology was implemented in [24] where a proposal of multi-optimization of surface roughness and productivity in dry turning of AISI 52100 steel using uncoated and PVD-coated cermet tools was carried out. The results of the predicted responses indicate that the feed rate is the most significant parameter and that the PVD coating has a better performance than the uncoated tool.

A statistical method to estimate the influence of cutting parameters on surface roughness in turning operations of several alloys of the Fe-C class and non-ferrous metals was proposed in [25]. Analytical and empirical relations describing the dependence of surface roughness in relation to feed rate were achieved and showed that the already well-known analytical relations of cutting parameters in the literature cannot always adequately describe the experimental data.

Das et al. [26] performed an investigation of cutting parameter influence on the dry turning of AISI 4340 steel using a CVD tool coated with multilayer TiN/TiCN/Al2O3/TiN. Experiments were designed using a factorial design technique with a Taguchi L9 orthogonal array and later performing an ANOVA. A highlight of the results is that the surface roughness is statistically influenced not only by the feed rate but also by the cutting speed, and that the CVD tool used for dry hard turning is effective and presents the potential to replace cylindrical grinding operations. The same AISI 4340 steel was evaluated for machinability according to wear and surface roughness criteria obtained in hard turning by Sahoo and Sahoo [27]. Three carbide tools were evaluated under the same cutting parameters: uncoated and two multilayer-coated inserts such as MTCVD (medium-temperature chemical vapor deposition) TiN, and conventional CVD ZrCN. First-and second-order linear regressions were proposed for the evolution of flank wear and surface roughness. ANOVA was used to statistically test the significance of second-order models and showed that multilayer-coated tools are more economically viable and that MTCVD is the best solution in turning surface finishing of hardened steel.

As previously presented, the development of new substrates and coating methods has demanded a constant reassessment of their influence on the performance of the metal cutting processes, such as cermet cutting tools. Aiming to contribute to the field, the purpose of this article is the assessment and optimization of surface roughness in turning of AISI 1045 steel, which is widely industrially applicable by using CVD- and PVD-coated carbides and PVD-coated cermet inserts. A design of experiments (DOE) with full factorial design with mixed levels to consider both quantitative and qualitative controllable factors in turning operations was used. Initially, a five-way ANOVA was used to verify the statistical significance of the main effects and the two-factor interaction effects of the cutting tool materials and coatings, the conditions of cutting parameters, and the tool nose radius on the surface roughness parameters, the arithmetic average height (*Ra*) and the ten-point height *Rz* (ISO) using MATLAB commercial software. Subsequently, the regression equations based on the RSM analysis, with the aid of Design Expert 12 commercial software, were proposed to predict the surface roughness parameters for each cutting insert.

## 2. Materials and Methods

### 2.1. Selection of Process Parameters (Controllable Factors) and Their Ranges

The process parameters that may affect the surface quality in turning processes have been identified based on a literature review. The quantitative process parameters such as cutting speed (*V_c_*), feed rate (*f*), depth of cut (DOC), tool nose radius (*r*) and the qualitative parameter of the insert substrate and coating method were selected as independent variables to analyze in this study, while the response variables were the surface roughness parameters. A computer numerical control (CNC) turning machine (ROMI CENTUR 30D, São Paulo, Brazil) which has variable spindle speed from 50 to 3500 rpm, a 9 kW motor drive and a Sinumerik 828D controller was used for dry machining of the workpieces, where the range of the controllable factors for turning experiments were selected based on technical recommendation and availability of cutting inserts from the tool manufacturer. The selection criterion for each number of categorical levels was defined according to the influence of its relationships already well established in the literature [28], according to which the feed rate and tool nose radius are usually the most significant parameters on machined surface roughness. To eliminate or minimize the effects of the fixation, each workpiece was fixed between a 3-jaw hydraulic chuck and tailstock of the CNC turning machine, and the run-out was verified, aiming to minimize it and assure similar turning conditions.

### 2.2. Material, Workpiece and Roughness Meter

**Figure 1:**
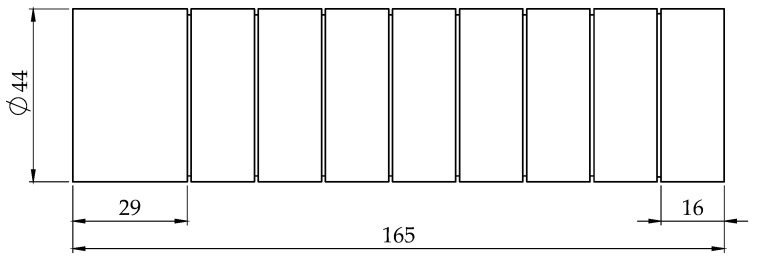
Profile and dimensions of the machined workpiece (units in mm).

Mild steel with designation AISI/SAE 1045 with a chemical composition in terms of mass of 0.43–0.5% C, 0.6–0.9% Mn, max. 0.040% P, max. 0.050% S and a hardness average of 178 HB was used for turning experiments. The workpieces used in the experiments had a cylindrical form, with 44 mm diameter and 165 mm length, as shown in Figure 1. Each 16 mm length of each workpiece was used for turning, with specific combinations of process parameters.

**Figure 2:**
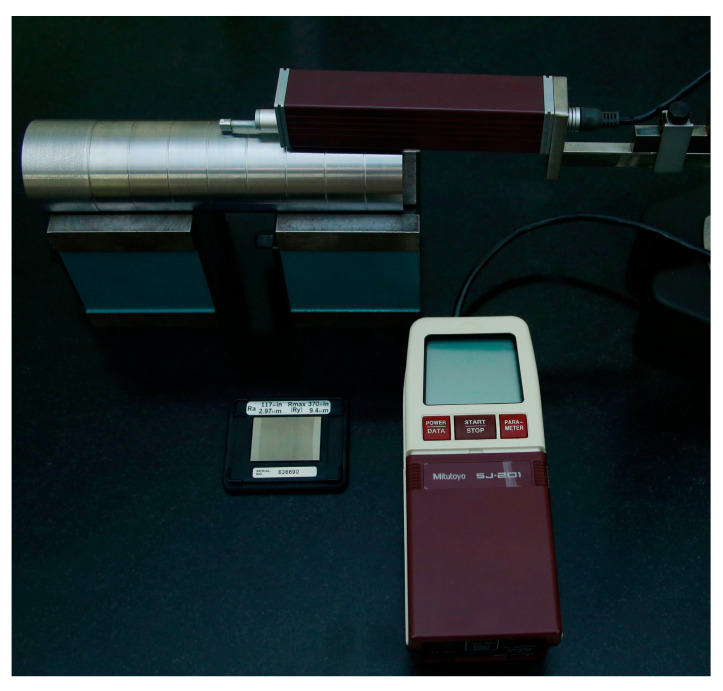
Setup for surface roughness measurement.

Measurement of the arithmetic average height *Ra* and the ten-point height *Rz* were carried out by a Mitutoyo roughness meter, model SJ-201, where the cutoff length and evaluation length are 0.8 mm and 4 mm, respectively. The instrument is initially calibrated using a standard calibration block with *Ra* roughness of 2.97 µm and the largest peak-to-valley height *Ry* of 9.4 µm. Two surface samples were turned for each combination of parameters, and the measurement of the workpiece surfaces (16 mm in length) was repeated three times in the axial lines direction equally positioned at 120 degrees around the cylindrical surface. Thus, all *Ra* and *Rz* average values were calculated by using six measurements from the roughness meter. Figure 2 illustrates the apparatus setup used to measure the workpieces surface roughness.

**Figure 3:**
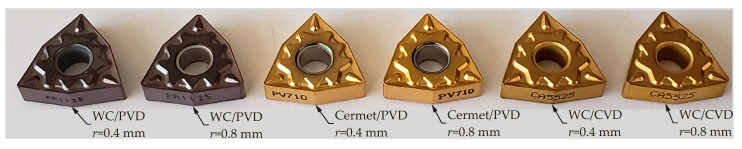
Turning inserts used in the experiments.

The inserts used in the experiments have ISO designations WNMG080404 and WNMG080408 (80° nose angle) with the same HQ chipbreaker specification. Three grades of cutting tools were used: Sintered tungsten carbide (WC) grade CA5525 (CVD coated TiCN + Al2O3 + TiN), and grade PR1125 (PVD coated TiAlN), and cermet grade PV710 (PVD coated TiCN). The inserts were clamped mechanically on a tool holder with ISO designation MTJNR2525M16. Figure 3 shows the substrates, coating methods, and geometries of the inserts used in the experiments.

### 2.3. Response Surface Methodology

The RSM technique is practical, economical, and relatively easy to use [29]. Generally, a low-order polynomial in some region of the independent variables space is appropriated, and if there is curvature in the response, then a higher-order polynomial must be used to propose a regression equation that can represent the relationship between the response and the independent variables in the space that includes the levels of controllable factors. The polynomial function that represents an approximation for the true response as a function of the input variables is shown in Equation (1), where *y* is the response variable, *x_i_* is the controllable factors, *β_0_*, *β_i_*, *β_ii_*, *β_ij_* are coefficients to be estimated and *k* is the number of controllable factors considered in the analysis [29]. After the mathematical modeling, the ANOVA is used in order to verify the equation terms’ significance.

(1) $$y=\beta_{0}+\sum_{i=1}^{k}\beta_{i}x_{i}+\sum_{i=1}^{k}\beta_{ii}x_{i}{}^{2}+\sum_{i<j}\sum \beta_{ij}x_{i}x_{j}+\varepsilon$$

This technique has been used by researchers to assess surface roughness parameters or other response variables in machining processes. The last step of RSM is the optimization of the response variables, which aims to determine the optimal machining conditions for a desired value of the response variable or to determine a region of the factorial space where the technical requirements are met. The optimization of the machining conditions is unknown before the experiments are performed, making RSM an important technique capable of obtaining optimal values of the response variables, considering a constrained optimization problem—in other words, according to the restricted goals of the process parameters.

### 2.4. Design of Experiments Using Factorials with Mixed Levels

The statistical design of the experiments deals with the process of planning the experiment so that the appropriate data are collected and analyzed by statistical methods, resulting in effective and objective conclusions. The statistical approach to the design of the experiments is necessary to produce significant conclusions about the dataset, so there are two aspects to any experimental problem: the design of the experiment and the statistical analysis of the data set [29]. There is a close interaction between the design of the experiments and the analysis of the regression model.

The controllable factors involved in an experiment can be quantitative or qualitative. A quantitative factor is one whose levels can be associated with values on a numerical scale, while the qualitative factor levels cannot be organized in terms of order of magnitude. A full factorial design consists of carrying out all possible combinations of discrete factor levels. Examples of full factorials for analyzing surface roughness or other response variables can be found in [20,30,31]. When a large number of factors are studied, a fractional factorial design can be used to keep the experiment at a reasonable size [26,32,33]. There is special interest in the design of experiments in which the number of levels of controllable factors differs, that is, mixed levels. If these are full factorials, then the construction and analysis of these designs present no new difficulties [29]. In this study, each of the process parameters is defined in discrete quantitative and qualitative levels, as shown in Table 1; therefore the design consists of 96 runs (2*2*3*2*4) of experiments performed based on the combination of the process parameters (or controllable factors).

### 2.5. Analysis of Variance

ANOVA can be done using statistical software, and in this work, the analysis was performed by commercial software MATLAB^®^ (version 2020, Mathworks, Natick, MA, USA) using the functions *anovan*, *interactionplot*, *multivarichart*, *maineffectsplot*, and *multcompare* to analyze the dominant significant effects, display two-factor interaction effects on the response variable to assess how the interactions affect the relationship between the factors and the response variable, graphically represent the relationships between factors and a response (especially useful in understanding interactions), test how response means are related to levels of one or more factors, and present which group means are significantly different, respectively. The significance level is usually based on a 95% confidence interval (*p*-value of F calculated < 5%). Analysis of variance is also used as one of the steps for applying RSM to verify parameters that need to be included in the mathematical model to predict the response variable. This is useful to test the significant controllable main factors and their interactions in the proposed regression equations for the response characteristics for each type of cutting tool employed in the experiments, so the insignificant coefficient terms (detected from ANOVA) can be omitted from the equations. A further process optimization step is proposed, based on goal criteria of surface roughness and productivity [34], which is carried out using the statistical commercial software Design Expert Version 12 (Stat-Ease, Minneapolis, MN, USA).

## 3. Results and Discussion

### 3.1. Experiment Results

The results of the full factorial plan developed for turning experiments are tabulated in Table 2, showing combinations of the controllable factors used for each run of experiment and the corresponding responses of *Ra* and *Rz* surface roughness parameters in terms of its average values. The type of substrate and coating method is a process parameter that has no value and therefore was considered a qualitative or categorical factor (see Table 1). The results of the experiment presented in Table 2 were used for the following analyses:

- a five-way ANOVA using a two-factor interactions model;
- regression model and statistical analysis;
- response surface methodology;
- optimization of parameters for lowest surface roughness.

### 3.2. Five-Way ANOVA

The analysis of variance was employed to detect significant controllable process factors in the multi-factor model presented in Table 1. The main effects on the means of the response variable (surface roughness parameters *Ra* and *Rz* presented in Table 2) for different levels of individual factors and their interactions are statistically tested for significant differences in group means. Table 3 and Table 4 show the significance results for a five-way ANOVA employing a two-factor interaction model, which can compute the *p*-values for null hypotheses on the main effects and the two-factor interaction effects or, in other words, compute whether the group means are significantly different considering the 95% confidence bounds. It can be noted that in Table 3, the main factor effects on the *Ra* surface roughness parameter in decreasing order of significance levels are the feed rate, tool nose radius, Substrate/Coating, DOC, and cutting speed. A similar analysis is shown in Table 4 where the decreasing order of significance levels for the *Rz* surface roughness parameter is feed rate, tool nose radius, DOC, Substrate/Coating, and cutting speed.

Figure S1 (Supplementary Material) shows the main effects plot for the five categorical factors according to Table 1. Differences are noted between mean levels for all factors. Further, the feed rate causes the strongest variation on the means of the *Ra* surface roughness parameter followed by tool nose radius, Substrate/Coating, DOC and small slope gradient for cutting speed effect. The parameter response *Rz* shows similar behavior, and the only difference in the order is that DOC is the third and the Substrate/Coating is the fourth. The response roughness parameters align with the theoretical geometric surface roughness as a function of the tool nose radius and the feed rate established by [28]. These plots also confirm the results shown in Table 3 and Table 4 evaluating the significant differences between group means for turning with different substrates and coating methods. In general, the sintered WC coated with the CVD method presents better performance, reaching high surface quality for the range of cutting parameters analyzed in this work, but the interaction effects must be evaluated.

**Figure 4:**
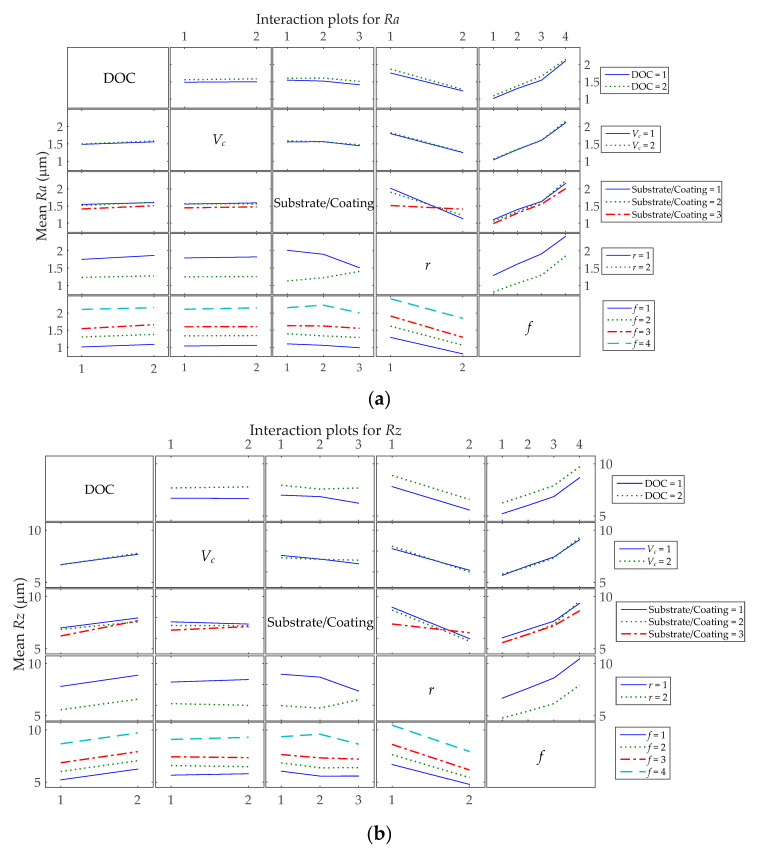
Interaction plots for surface roughness means: (**a**) *Ra*; (**b**) *Rz*.

**Figure 5:**
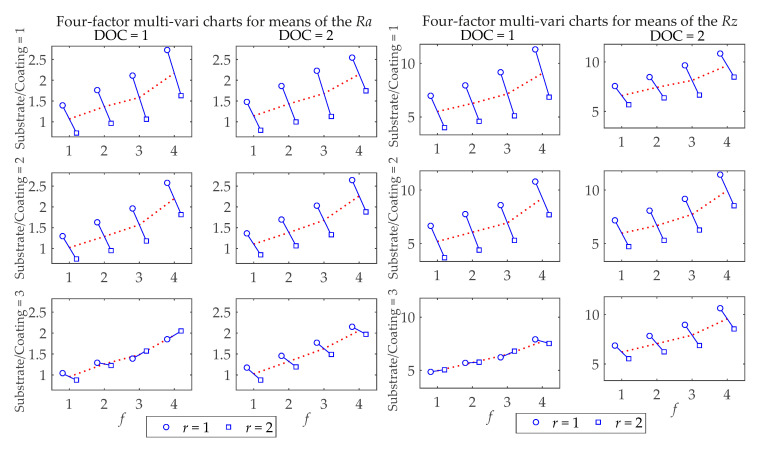
Four-factor multi-vari chart for *Ra* and *Rz* surface roughness means.

**Figure 6:**
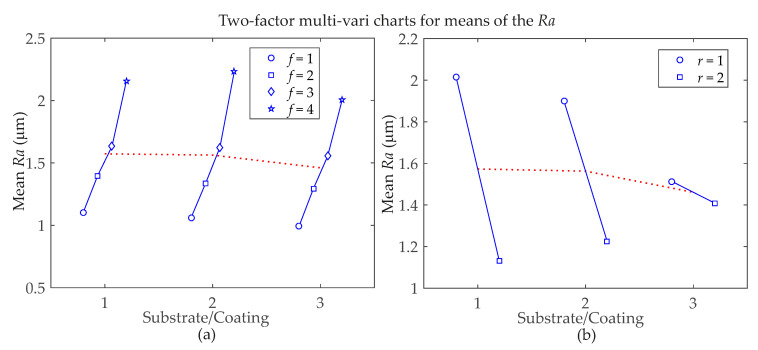
Two-factor multi-variable charts for *Ra* surface roughness parameter means, considering interactions between: (**a**) Substrate/Coating with feed rate; (**b**) Substrate/Coating with nose radius.

**Figure 7:**
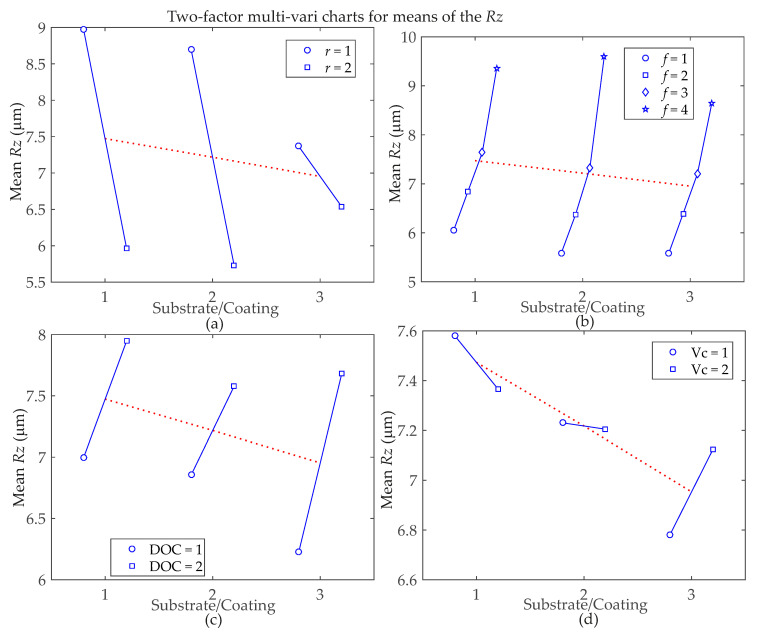
Two-factor multi-vari charts for *Rz* surface roughness parameter means, considering interactions between: (**a**) Substrate/Coating with nose radius; (**b**) Substrate/Coating with feed rate; (**c**) Substrate/Coating with depth of cut; (**d**) Substrate/Coating with cutting speed.

Figure 4 displays the two-factor interaction plot for the five main process factors, which evidence the interaction effects; in other words, the relationship between one categorical factor and the continuous surface roughness parameters depends on the second categorical factor. It can be noted from Table 3 and Table 4 that the most dominant two-factor interaction effects on both surface roughness parameters are Substrate/Coating*r. The interactions are considered significant based on a 95% confidence bound. Regarding the *Ra* surface roughness, it is shown in Figure 4a that most interactions show lines that are almost parallel, so small interaction effects occur, but there are different behaviors for Substrate/Coating*r and Substrate/Coating*f interactions. The first interaction effect shows that the relationship between the nose radius and the roughness parameter depends on the Substrate/Coating. For example, if categorical factor *r* = 1 is used, Substrate/Coating = 3 is associated with the lowest mean roughness. However, if *r* = 2 is used, Substrate/Coating = 1 is associated with the lowest mean roughness. The second interaction effect indicates that for *f* = 4, the lowest roughness is achieved for workpieces turned by Substrate/Coating = 3, and it increases when Substrate/Coating = 2 is used and decreases when Substrate/Coating = 1 is used. Nonetheless, for the *Rz* surface roughness, the analysis of Figure 4b shows that the main interaction effects take place between Substrate/Coating with *r*, *f*, *V_c_* or DOC. The best combinations for the lowest mean of the *Rz* are as follows: *r* = 1 with Substrate/Coating = 3; *r* = 2 with Substrate/Coating = 2; *f* = 4 with Substrate/Coating = 3; *f* = 1 with Substrate/Coating = 2. The Substrate/Coating 1 and 2 have lower mean *Rz* values for *V_c_* = 2. However, if Substrate/Coating = 3 is used, the *V_c_* = 1 produces lower mean *Rz* values. These interaction effects also can be seen in Figure 5, Figure 6 and Figure 7 in more detail.

Figure 5 shows the multi-vari charts with the main significant factors for *Ra* and *Rz* surface roughness means. It can be observed in Figure 6 that for the most significant process factor (*f*), the higher the feed rates are, the higher *Ra* and *Rz* are. It can be noticed that the variation in *Ra* is lower when turning with Substrate/Coating = 3 than with other cutting tools. If *r* = 2 is used, *Ra* is lower when turning with Substrate/Coating = 1; however, if *r* = 1 is used, *Ra* tends to be lower with Substrate/Coating = 3. In addition, Figure 5 illustrates that Substrate/Coating = 3 has better performance (lower mean *Rz* values) except for the combinations DOC = 2, *r* = 2 and DOC = 1, *r* = 2, for which Substrate/Coating = 2 has better performance when it comes to workpieces’ surface roughness.

Figure 6 and Figure 7 show a graphical representation of the relationships between factors and *Ra* and *Rz* surface roughness responses, respectively, for a better understanding of two-factor interaction effects. There are four charts for *Rz* in Figure 7 because, according to Table 4, there are more significant interactions for this response parameter. Considering the *Ra* roughness parameter means in Figure 6, the Substrate/Coating*f interaction shows that the Substrate/Coating = 3 performs better regardless of the level of the feed rate (*f*). For Substrate/Coating*r interaction, the combinations with the lowest *Ra* means are achieved by using *r* = 2 with Substrate/Coating = 1, but when *r* = 1 is necessary, Substrate/Coating = 3. For *Rz* roughness parameter means, Figure 7 shows that for the Substrate/Coating*r interaction, the best results are obtained when *r* = 1 with Substrate/Coating = 3, but when *r* = 2 is necessary, Substrate/Coating = 2. Considering the Substrate/Coating*f interaction for the highest feed rate level, *f* = 4, the best mean results are achieved with Substrate/Coating = 3. However, for the smallest feed rate level, *f* = 1, Substrate/Coating = 2 performs better. In the Substrate/Coating*DOC interaction, when DOC = 1, the best option is Substrate/Coating = 3; Moreover, a narrow variation is observed in the *Rz* means when Substrate/Coating = 2 is used. For the Substrate/Coating*V_c_ interaction, when Substrate/Coating = 1 or Substrate/Coating = 2 is used, the best cutting speed is *V_c_* = 2, but when using Substrate/Coating = 3, the best cutting speed is *V_c_* = 1. In addition, for the latter, the smallest variation in the roughness means is achieved by using Substrate/Coating = 2.

Figure S2 (Supplementary Material) shows the multiple comparison of group means. Figure S2a,c illustrates the group means of the resulting workpieces surface roughness using different cutting inserts, demonstrating that WC/CVD has better performance for both response variables within the range interval of controllable factors shown in Table 1, and the *Ra* group means for workpieces turned by using cermet/PVD compared to WC/PVD have overlapping intervals, so they are not significantly different. Figure S2b,d (Supplementary Material) displays that the group means of the resulting workpieces surface roughness using different cutting speeds are significantly different for *Ra* because the intervals are disjointed but are not for *Rz* as the intervals overlap, considering 95% confidence bounds.

### 3.3. Regression Model and Statistical Analysis

Firstly, the RSM was used to perform a regression analysis to find an adequate fitting model that can assess the behavior of the *Ra* and *Rz* response variables as a function of the significant independent variables shown in Table 1, and the final objective was to optimize the responses. The polynomial models, such as the linear model, the two-factor interaction model (2FI) and the quadratic model, were statistically assessed. The results for both *Ra* and *Rz* responses are presented in Table 5 and Table 6, respectively. This step was used to select a correct model according to the following criterion: the highest polynomial order but not aliased. The quadratic model has the highest polynomial order, but it is aliased. This indicates that the model cannot be accurately fitted to the design of experiments and should not be selected for analysis. The focus is also to maximize the adjusted R² and the predicted R² for the selected model.

A 2FI model that includes linear and two-factor interaction terms was selected to describe an approximate polynomial relationship between the process parameters shown in Table 1 and the *Ra* and *Rz* roughness parameters. The initial analysis of the responses includes all process parameters and their two-factor interactions. The analysis of variance in this case is used to determine which process parameters and two-factor interactions significantly affect the responses. The smaller the *p*-values (Prob > F) are, the more significant the results are, indicating that the terms are significant and can improve the fitting of the response surfaces. Table 7 and Table 8 show these results of ANOVA for *Ra* and *Rz*, respectively. This analysis is performed by considering that *p*-values lower than 0.05 indicate that model terms are significant, and values greater than 0.10 indicate that model terms are not significant. For the *Ra* and *Rz* response variables, the main effects and interactions (A-DOC, C-*r*, D-*f*, E-Substrate/Coating, DOC*r, r*f, Substrate/Coating*r, Substrate/Coating*f) and (A-DOC, C-*r*, D-*f*, E-Substrate/Coating, DOC*Substrate/Coating, V_c_*Substrate/Coating, r*f, Substrate/Coating*r, Substrate/Coating*f) are significant model terms, respectively. A model may be improved by reducing insignificant terms, excepting those required to support hierarchy. For the *Rz* response, if the interaction term V_c_*Substrate/Coating is statistically significant, making the main effect B-*V_c_* theoretically relevant even if not statistically significant, then factor B-*V_c_* is considered in the regression equation.

The regression equations for the response variables (*Ra* and *Rz*) for each Substrate/coating as a function of the process parameters considered in these experiments are given in Equations (2)–(7). The insignificant coefficient terms established by ANOVA have been eliminated from the equations. These equations in terms of current controllable factors (*V_c_* in mm/min, *f* in mm/rev., DOC in mm, *r* in mm) can be used to make predictions about the responses for given levels of each factor.

(2) $$R_{a(\mathrm{WC}/\mathrm{PVD})}=0.056286+0.469167\mathrm{DOC}-1.29966r+16.56429f-0.477083\mathrm{DOC}r-4.1756rf$$

(3) $$R_{a(\mathrm{WC}/\mathrm{CVD})}=-1.06462+0.469167\mathrm{DOC}+0.740967r+15.05f-0.477083\mathrm{DOC}r-4.1756rf$$

(4) $$R_{a(\mathrm{Cermet}/\mathrm{PVD})}=-0.432357+0.469167\mathrm{DOC}-0.680908r+17.13214f-0.477083\mathrm{DOC}r-4.1756rf$$

(5) $$R_{z(\mathrm{WC}/\mathrm{PVD})}=2.14446+2.1675\mathrm{DOC}-0.00345V_{c}-4.12354r+59.16071f-24.04167rf$$

(6) $$R_{z(\mathrm{WC}/\mathrm{CVD})}=-2.74771+2.8825\mathrm{DOC}+0.003312V_{c}+1.68271r+52.23929f-24.04167rf$$

(7) $$R_{z(\mathrm{Cermet}/\mathrm{PVD})}=0.538393+1.45\mathrm{DOC}-0.00025V_{c}-3.68917r+65.10714f-24.04167rf$$

**Figure 8:**
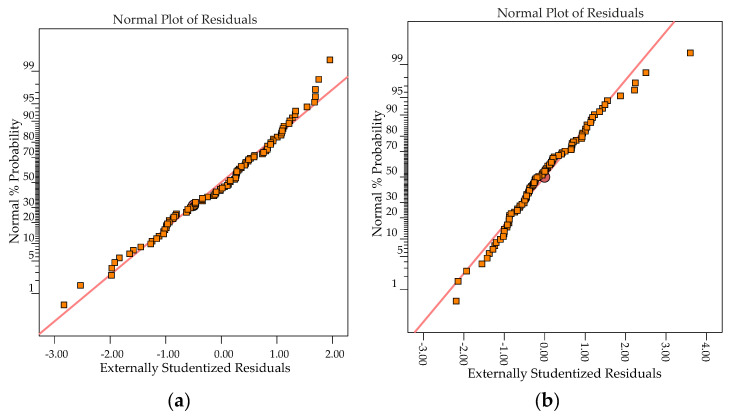
Normal plot of residuals: (**a**) *Ra*; (**b**) *Rz*.

**Figure 9:**
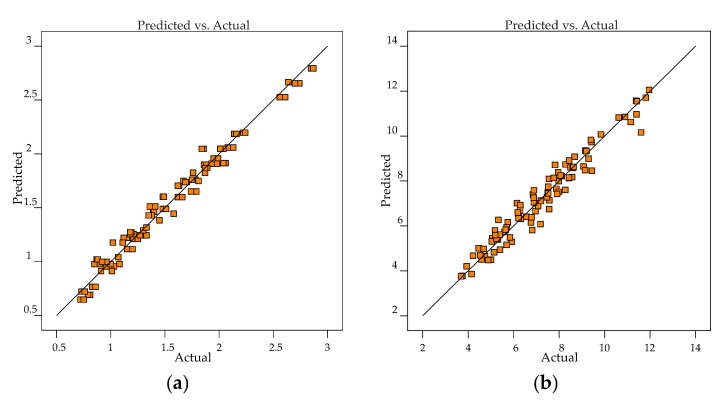
Predicted versus actual response values: (**a**) *Ra*; (**b**) *Rz*.

The regression equations were assessed to verify if the fit of the model is adequate. Residuals are the deviation between predicted and actual values, and they should follow a normal distribution if the experimental errors are random. Figure 8 shows the normal plot of residuals for *Ra* and *Rz* responses. It can be noted that for responses, the residuals are approximately along a straight line, and hence the studentized residuals follow a normal distribution. The predicted vs. actual graph helps to detect observations that are not well predicted by the regression equations (Figure 9). As the data points are evenly split by the 45-degree line, it implies that the regression model is adequate. 

### 3.4. Response Surface Analysis

**Figure 10:**
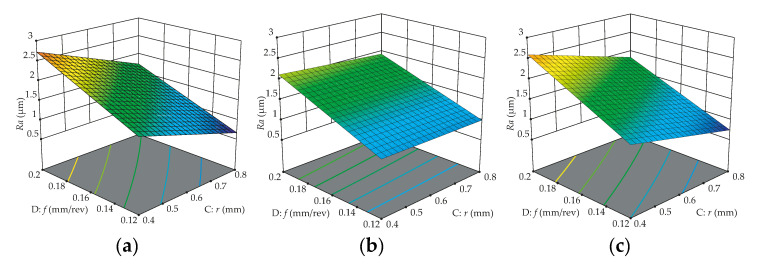
Predicted effect of feed rate and nose radius on *Ra* surface roughness for cutting tools: (**a**) WC/PVD; (**b**) WC/CVD; (**c**) Cermet/PVD.

**Figure 11:**
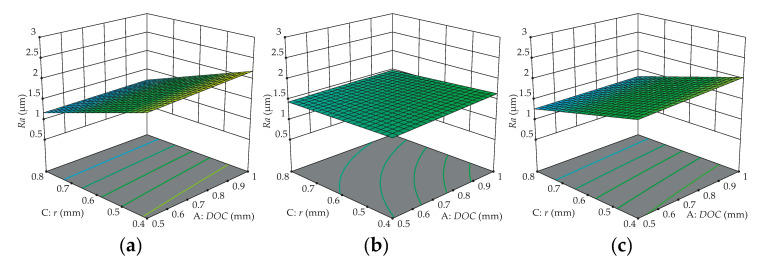
Predicted effect of nose radius and depth of cut on *Ra* surface roughness for cutting tools: (**a**) WC/PVD; (**b**) WC/CVD; (**c**) Cermet/PVD.

**Figure 12:**
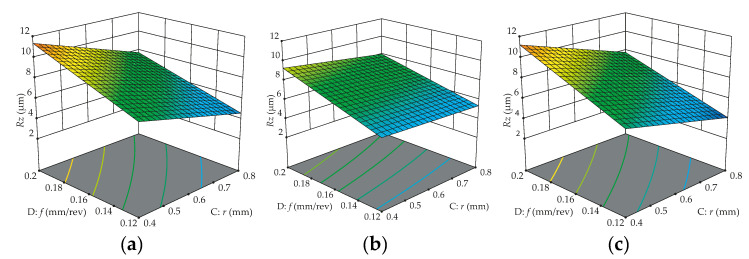
Predicted effect of feed rate and nose radius on *Rz* surface roughness for cutting tools: (**a**) WC/PVD; (**b**) WC/CVD; (**c**) Cermet/PVD.

To verify the influences of controllable factors on the surface roughness parameters, response surfaces are shown in Figure 10, Figure 11 and Figure 12, describing the significant interaction effects of different variables on responses. All fixed parameters are set at the middle level or according to what is illustrated in the figures. As can be noticed in Figure 10, the *Ra* surface roughness is more stable when WC/CVD is used; in other words, there is lower variation on *Ra* when the workpiece is turned with this type of cutting tool. Figure 10 also shows that the feed rate has more statistical importance than the nose radius on surface roughness (*Ra*), regardless of the cutting tool type used, in concordance with the literature [28]; i.e., the best surface roughness is achieved for higher nose radii with lower feed rates.

Figure 11 also demonstrates that WC/CVD has lower variation on *Ra* surface roughness, and the depth of cut has a small significance of response, consequently showing a small slope gradient for all types of cutting tools. A different behavior can be noticed when the workpiece is turned with WC/CVD, where the nose radius also has a small slope gradient, showing that it also has low significance when this insert is used. In Figure 12, the predicted response surface of roughness *Rz* as a function of feed rate and nose radius is depicted. These surface plots confirm the significant influences of two controllable factors and show that the WC/CVD insert produces lower variations on *Rz*. The best *Rz* surface roughness also is achieved for higher nose radii with lower feed rates. 

### 3.5. Optimization of Parameters for the Lowest Surface Roughness

After the important factors for the response variables were identified, the next objective was to find the optimal or near-optimal settings or levels of the important factors that result in desirable values of the responses. The first optimization was executed targeting the lowest surface roughness, and the second was performed to find the optimal controllable factors to turn the workpiece with lower roughness and the highest material removal rate. The goals with the parameter ranges defined for the first optimization process for *Ra* and *Rz* are summarized in Table 9. Table 10 and Table 11 present six predicted optimization results, considering a decreasing desirability for *Ra* and *Rz*, respectively.

**Figure 13:**
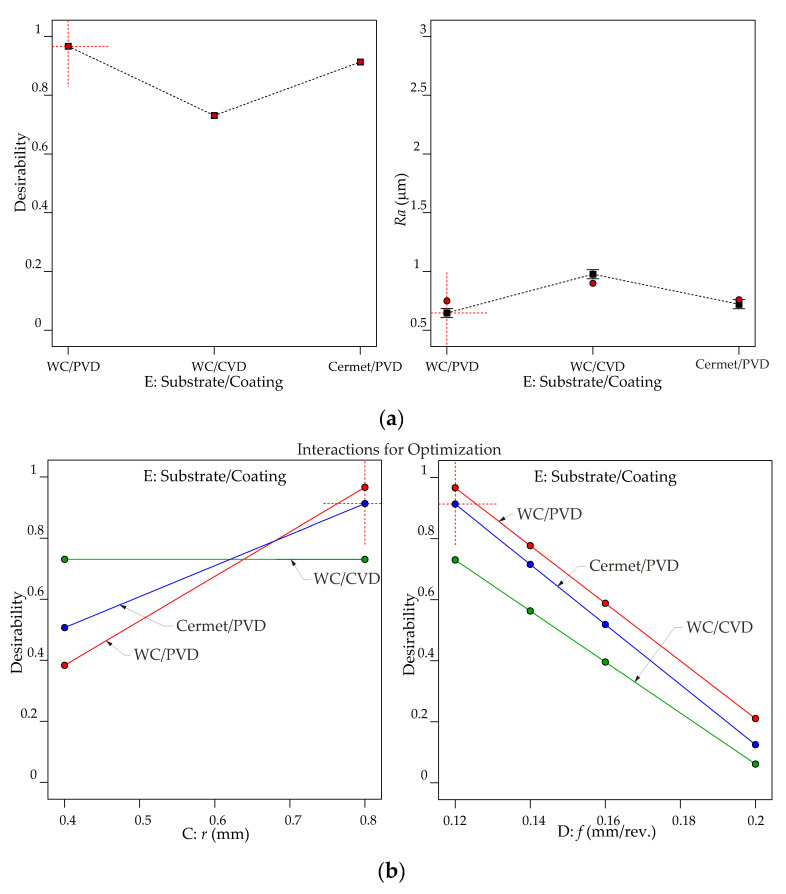

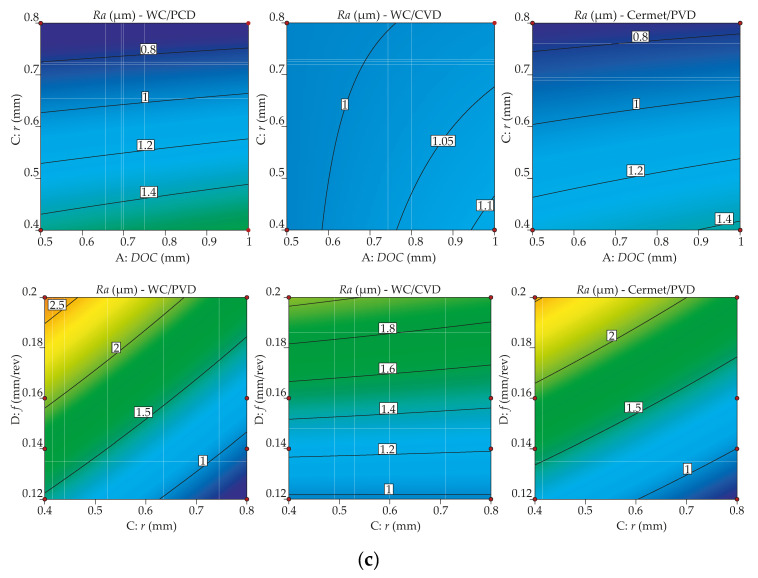
Graphs of optimization results to minimize *Ra* as goal: (**a**) effect of cutting tool type; (**b**) interaction effects of Substrate/Coating*r and Substrate/Coating*f; (**c**) *Ra* contour levels of interactions DOC*r and r*f.

**Figure 14:**
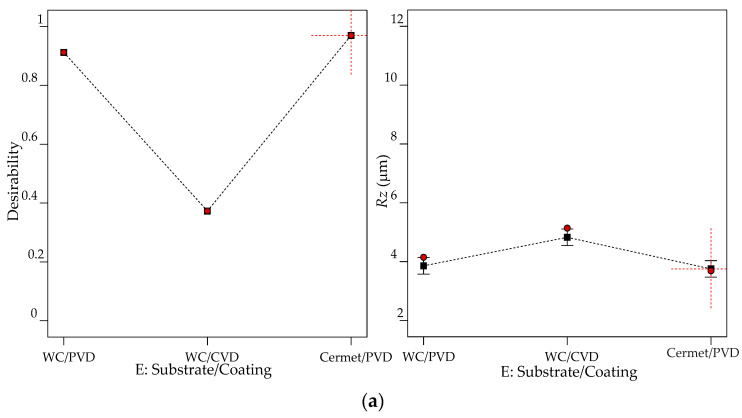

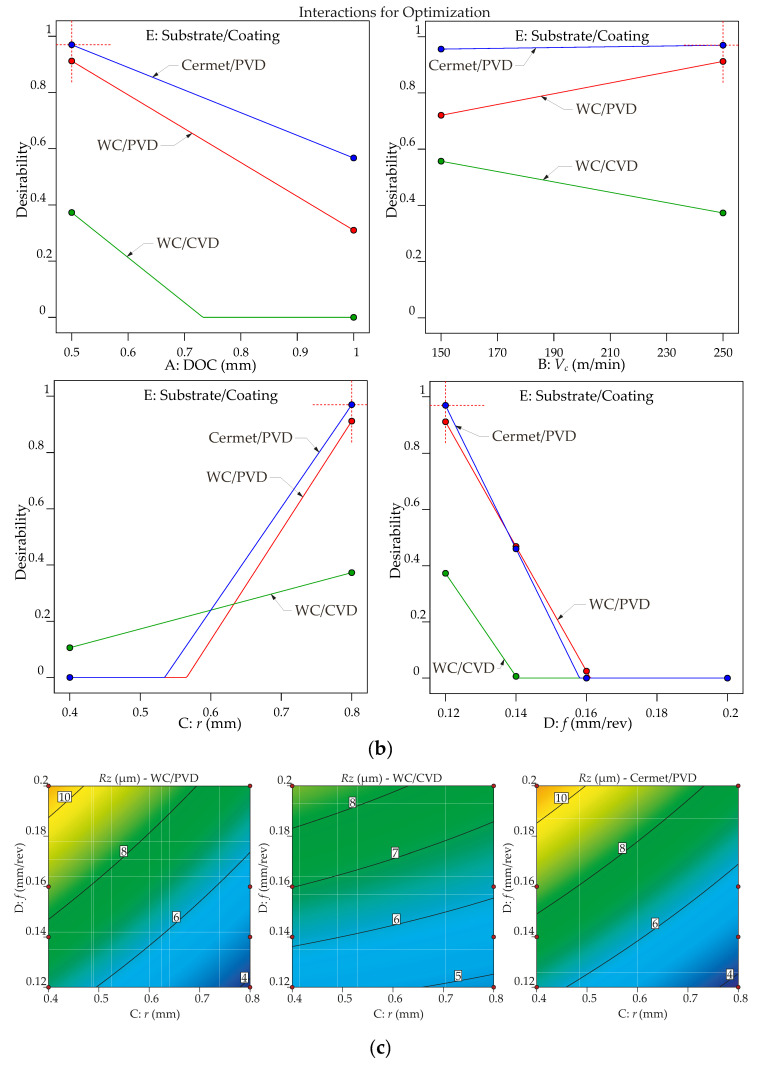
Graphs of optimization results to minimize *Rz* as a goal: (**a**) effect of cutting tool types; (**b**) interaction effects of DOC*Substrate/Coating, V_c_*Substrate/Coating, Substrate/Coating*r and Substrate/Coating*f; (**c**) *Rz* contour levels versus nose radius and feed rate.

Figure 13 and Figure 14 illustrate graphs of optimization results for *Ra* and *Rz*, respectively. The controllable factors that are not shown in each graph are set according to the first optimized solution shown in Table 10 and Table 11, respectively. Figure 13a demonstrates that to achieve the lowest *Ra* and consequently the highest desirability, the WC/PVD insert must be used. Cermet/PVD insert is one of the optimized options in Table 10; however, its desirability is lower than that of WC/PVD. In addition, the WC/CVD insert cannot be used when the only target is to reach the best surface quality. In Figure 13b, the interaction effects r*f and Substrate/Coating*f show that all the optimized solutions use radii equal to 0.8 mm and 0.12 mm/rev. feed rate; therefore, only WC/PVD and cermet/PVD inserts can obtain a reasonable desirability. Figure 13c shows predicted *Ra* contour levels of interactions DOC*r and r*f, which also demonstrate that the best combination is the lowest DOC and feed rate with the highest nose radius.

Figure 14a shows that cermet/PVD is the best cutting tool when the lowest *Rz* is the goal. Figure 14b illustrates the behavior of desirability resulting from the interactions of cutting tool types with the other controllable factors. Figure 14c shows predicted *Rz* contour levels as a function of the nose radius and feed rate and demonstrates that the best combination is the lowest feed rate and the highest nose radius.

**Figure 15:**
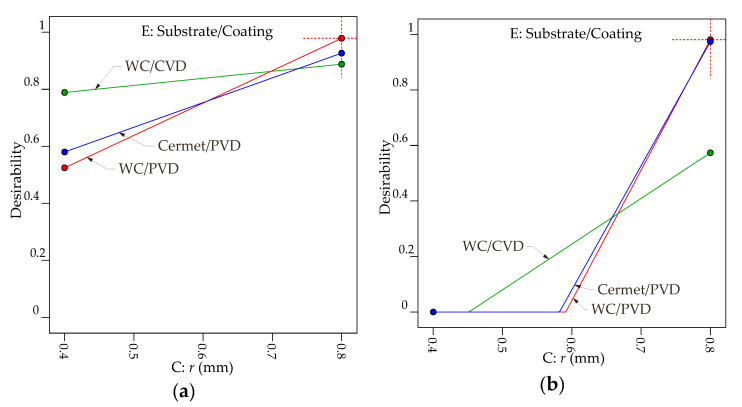
Graphs of optimization results to minimize roughness parameters with the maximum removal rate: interaction effects between Substrate/Coating*r for (**a**) *Ra*, (**b**) *Rz*.

The second optimization is performed with the target to indicate the combinations of the controllable factors generating the lowest roughness with the highest material removal rate. The goals with the parameter ranges defined for the optimization process for both removal rate and roughness parameters *Ra* and *Rz* are summarized in Table 12. Table 13 and Table 14 present six and three predicted optimization results considering a decreasing desirability for *Ra* and *Rz*, respectively. Figure 15 illustrates the results for the maximum removal rate minimizing the *Ra* and *Rz*.

Figure 15a demonstrates that interaction effects between Substrate/Coating*r indicate that the best desirability of 0.979 is obtained when the nose radius is equal to 0.8 mm and the WC/PVD is used in the turning process. If a 0.4 mm nose radius is used, the WC/CVD has the highest desirability, 0.789. A similar conclusion can be seen in Table 13, which shows the possible solutions with decreasing desirability. Figure 15b shows that only the WC/PVD and cermet/PVD inserts can achieve reasonable desirability, 0.981 and 0.974 respectively, and only the application of a 0.8 mm nose radius indicates that it is possible to obtain the desired solutions. Therefore, the possibilities considering the highest removal rate combined with a surface quality criteria are more limited for *Rz* than for *Ra*.

## 4. Conclusions

In this study, the influence of machining parameters such as cutting speed, depth of cut, tool nose radius, feed rate and the substrate and coating method of cutting tools on surface roughness is investigated in turning of AISI 1045 steel, which is widely industrially applicable. Few studies have analyzed the effects of carbide and cermet cutting inserts or the two most common available coating processes on surface roughness. The analysis was preceded by a five-way ANOVA to determine the significant main effects and two-factor interaction effects on the *Ra* and *Rz* roughness parameters. In addition, the application of RSM was used to obtain mathematical models (2FI) for both *Ra* and *Rz* surface roughness parameters as a function of the significant turning parameters and their interactions for each cutting insert. Optimum values of turning parameters have been obtained considering either the highest surface quality or the lowest surface roughness combined with the highest material removal rate. The main conclusions drawn are as follows:

- The specific lowest surface roughness for the *Ra* parameter is obtained by using WC/PVD inserts, but the group mean roughness of the turned workpieces obtained using WC/CVD inserts was lower than the group means of other types of inserts. In other words, this could be the most versatile insert when the manufacturing processes need to use any combination within the range interval of controllable factors.
- There are some significant two-factor interaction effects that achieve the lowest *Ra* and *Rz* roughness parameters. Considering the *Ra* parameter, the interaction effects are between Substrate/Coating with *r* or *f*, and for *Rz* Substrate/Coating with *r*, *f*, DOC or *V_c_*.
- Both *Ra* and *Rz* roughness parameters always have higher values for turned workpieces using higher feed rates and lower nose radii, confirming that they follow the theoretical equations for the geometric surface roughness in turning processes.
- The response optimization for the predicted lowest *Ra* surface roughness parameter of 0.647 µm and a desirability of 0.966 is obtained by using WC/PVD inserts with *DOC* = 0.5 mm, *V_c_* = 250 m/min, *r* = 0.8 mm and *f* = 0.120 mm/rev. However, when considering the *Rz* surface roughness parameter, the response optimization infers that the cermet/PVD outperforms the other cutting inserts, with a predicted value of 3.754 µm and desirability of 0.970.
- For the target of the best surface finish combined with a maximum material removal rate of 40 cm^3^/min, the response optimization shows that the most suitable inserts would be WC/PVD, followed very closely by Cermet/PVD, with the lowest predicted values for *Ra* = 1.749 µm and *Rz* = 8.136 µm and desirability of 0.979 and 0.981, respectively.
- Finally, the response optimization shows that, within the range interval of controllable factors used in this analysis and the surface finish criteria, the fine adjustment of the turning process can improve productivity and also be an alternative to grinding operations, since the predicted surface finish values for *Ra* parameter are under 2 µm.

## Figures and Tables

**Table 1:** Quantitative and qualitative controllable factors and respective mixed levels used for a full factorial DOE.

Parameters	Symbol	Unit	Categorical Levels			
			1	2	3	4
Depth of cut	DOC	mm	0.5	1.0	-	-
Cutting speed	*V_c_*	m/min	150	250	-	-
Substrate/Coating method	-	-	WC ^1^/PVD	Cermet/PVD	WC/CVD	-
Nose radius	*r*	mm	0.4	0.8	-	-
Feed rate	*f*	mm/rev	0.12	0.14	0.16	0.20

^1^ WC: Sintered Tungsten Carbide.

**Table 2:** Average values of experimental results for surface roughness parameters.

Run	Factor 1	Factor 2	Factor 3	Factor 4	Factor 5	Average Values for Surface Roughness Parameters	
	A:DOC	B:*V_c_*	C:*r*	D:*f*	E:Substrate/Coating	*Ra*	*Rz*
	mm	m/min	mm	mm/rev	Categorical	µm	µm
01	1.0	150	0.8	0.20	Cermet/PVD	1.89	8.57
02	0.5	250	0.8	0.12	Cermet/PVD	0.76	3.69
03	0.5	150	0.4	0.20	WC/PVD	2.74	11.40
04	1.0	250	0.4	0.20	WC/CVD	2.14	11.80
05	0.5	250	0.8	0.20	WC/CVD	2.04	7.50
06	0.5	150	0.8	0.12	Cermet/PVD	0.73	3.75
07	0.5	150	0.4	0.14	WC/CVD	1.27	5.65
08	1.0	250	0.4	0.12	Cermet/PVD	1.35	7.20
09	0.5	250	0.4	0.12	Cermet/PVD	1.30	6.79
10	1.0	150	0.8	0.20	WC/CVD	1.95	8.61
11	0.5	250	0.8	0.20	WC/PVD	1.62	6.89
12	0.5	150	0.4	0.12	Cermet/PVD	1.30	6.56
13	1.0	150	0.4	0.12	WC/CVD	1.15	6.22
14	0.5	250	0.4	0.12	WC/CVD	1.08	5.04
15	1.0	250	0.4	0.20	Cermet/PVD	2.64	11.42
16	0.5	150	0.4	0.20	Cermet/PVD	2.61	10.90
17	0.5	250	0.4	0.20	WC/CVD	1.86	7.82
18	1.0	150	0.4	0.20	Cermet/PVD	2.64	11.38
19	0.5	250	0.4	0.16	Cermet/PVD	1.93	8.50
20	0.5	150	0.4	0.16	WC/PVD	2.13	9.29
21	1.0	150	0.8	0.12	WC/PVD	0.81	5.91
22	0.5	150	0.8	0.20	WC/PVD	1.63	6.85
23	1.0	150	0.8	0.20	WC/PVD	1.81	9.14
24	0.5	150	0.4	0.20	WC/CVD	1.84	7.96
25	0.5	250	0.8	0.12	WC/PVD	0.75	4.15
26	1.0	150	0.8	0.12	Cermet/PVD	0.83	4.59
27	0.5	250	0.8	0.16	WC/CVD	1.58	6.76
28	1.0	150	0.4	0.12	WC/PVD	1.48	7.55
29	0.5	250	0.8	0.12	WC/CVD	0.90	5.14
30	1.0	250	0.8	0.12	Cermet/PVD	0.86	4.88
31	1.0	250	0.8	0.20	Cermet/PVD	1.87	8.43
32	1.0	250	0.4	0.16	WC/PVD	2.24	9.42
33	1.0	250	0.4	0.12	WC/CVD	1.20	7.56
34	0.5	150	0.4	0.12	WC/CVD	1.00	4.68
35	1.0	150	0.4	0.20	WC/CVD	2.16	9.40
36	0.5	150	0.8	0.16	Cermet/PVD	1.19	5.39
37	0.5	250	0.4	0.14	WC/PVD	1.75	7.88
38	1.0	250	0.4	0.16	WC/CVD	1.79	9.44
39	0.5	250	0.4	0.14	WC/CVD	1.33	5.74
40	0.5	250	0.8	0.20	Cermet/PVD	1.87	7.91
41	0.5	150	0.4	0.16	WC/CVD	1.36	6.28
42	1.0	150	0.8	0.14	Cermet/PVD	1.07	5.25
43	1.0	150	0.4	0.16	WC/PVD	2.22	9.84
44	1.0	250	0.8	0.20	WC/CVD	1.99	8.44
45	1.0	150	0.8	0.14	WC/PVD	1.03	7.18
46	1.0	250	0.4	0.20	WC/PVD	2.87	11.81
47	0.5	150	0.8	0.12	WC/CVD	0.85	5.00
48	1.0	150	0.4	0.14	WC/PVD	1.86	8.68
49	0.5	150	0.8	0.16	WC/CVD	1.58	6.81
50	1.0	250	0.8	0.14	Cermet/PVD	1.07	5.31
51	0.5	250	0.8	0.16	WC/PVD	1.11	5.06
52	1.0	150	0.4	0.14	WC/CVD	1.45	7.46
53	0.5	150	0.8	0.14	WC/CVD	1.21	5.69
54	1.0	150	0.8	0.12	WC/CVD	0.87	5.69
55	1.0	250	0.8	0.16	Cermet/PVD	1.33	6.32
56	0.5	250	0.8	0.16	Cermet/PVD	1.18	5.19
57	0.5	250	0.4	0.20	Cermet/PVD	2.56	10.62
58	1.0	250	0.8	0.12	WC/CVD	0.88	5.33
59	1.0	150	0.8	0.16	Cermet/PVD	1.33	6.22
60	0.5	250	0.4	0.14	Cermet/PVD	1.62	7.51
61	0.5	250	0.8	0.14	WC/PVD	1.01	4.74
62	1.0	250	0.4	0.16	Cermet/PVD	2.01	9.20
63	0.5	250	0.4	0.20	WC/PVD	2.70	11.15
64	1.0	250	0.8	0.16	WC/CVD	1.51	6.89
65	1.0	250	0.8	0.14	WC/CVD	1.21	6.29
66	0.5	150	0.8	0.20	Cermet/PVD	1.76	7.52
67	1.0	250	0.8	0.12	WC/PVD	0.79	5.40
68	1.0	150	0.8	0.16	WC/CVD	1.48	6.88
69	0.5	250	0.4	0.12	WC/PVD	1.40	6.96
70	1.0	250	0.4	0.14	WC/PVD	1.88	8.28
71	0.5	250	0.4	0.16	WC/CVD	1.41	6.15
72	0.5	150	0.4	0.14	Cermet/PVD	1.66	7.99
73	1.0	150	0.8	0.14	WC/CVD	1.18	6.18
74	1.0	250	0.4	0.14	WC/CVD	1.45	8.27
75	0.5	150	0.4	0.12	WC/PVD	1.39	6.98
76	0.5	150	0.8	0.12	WC/PVD	0.72	3.93
77	0.5	250	0.8	0.14	Cermet/PVD	0.92	4.21
78	1.0	150	0.4	0.16	WC/CVD	1.74	8.43
79	0.5	250	0.8	0.14	WC/CVD	1.25	5.84
80	0.5	150	0.8	0.20	WC/CVD	2.06	7.57
81	1.0	250	0.4	0.12	WC/PVD	1.49	7.52
82	0.5	150	0.8	0.14	WC/PVD	0.91	4.46
83	1.0	150	0.4	0.12	Cermet/PVD	1.38	7.20
84	0.5	150	0.8	0.16	WC/PVD	1.02	5.18
85	1.0	250	0.4	0.14	Cermet/PVD	1.70	8.11
86	1.0	250	0.8	0.20	WC/PVD	1.67	7.76
87	0.5	250	0.4	0.16	WC/PVD	2.08	9.07
88	1.0	150	0.8	0.16	WC/PVD	1.15	7.08
89	1.0	150	0.4	0.20	WC/PVD	2.85	11.95
90	0.5	150	0.4	0.14	WC/PVD	1.78	7.98
91	1.0	150	0.4	0.14	Cermet/PVD	1.69	8.06
92	0.5	150	0.8	0.14	Cermet/PVD	0.96	4.53
93	1.0	250	0.8	0.16	WC/PVD	1.12	6.20
94	1.0	150	0.4	0.16	Cermet/PVD	2.04	9.15
95	1.0	250	0.8	0.14	WC/PVD	0.96	5.59
96	0.5	150	0.4	0.16	Cermet/PVD	1.98	8.63

**Table 3:** Standard five-way ANOVA table results for *Ra* applying two-factor interactions model.

Analysis of Variance						
Source	Sum Sq.^1^	d.f.^2^	Mean Sq.^3^	F Value	Prob > F	PCR ^4^ (%)
DOC	0.8875	1	0.8875	100.7004	0.0000	0.58%
*V_c_*	0.0512	1	0.0512	5.8080	0.0163	0.03%
Substrate/Coating	1.4731	2	0.7365	83.5694	0.0000	0.96%
*r*	44.1837	1	44.1837	5013.1965	0.0000	28.66%
*f*	90.7238	3	30.2413	3431.2512	0.0000	58.84%
DOC*V_c_	0.0050	1	0.0050	0.5626	0.4535	0.00%
DOC*Substrate/Coating	0.0514	2	0.0257	2.9139	0.0551	0.03%
DOC*r	0.1678	1	0.1678	19.0343	0.0000	0.11%
DOC*f	0.0875	3	0.0292	3.3077	0.0200	0.06%
Vc*Substrate/Coating	0.0453	2	0.0227	2.5710	0.0774	0.03%
V_c_*r	0.0129	1	0.0129	1.4681	0.2262	0.01%
V_c_*f	0.0317	3	0.0106	1.1991	0.3094	0.02%
Substrate/Coating*r	15.5593	2	7.7797	882.6991	0.0000	10.09%
Substrate/Coating*f	0.5585	6	0.0931	10.5607	0.0000	0.36%
r*f	0.3502	3	0.1167	13.2450	0.0000	0.23%
Error	4.7857	543	0.0088	-	-	-
Total	158.9745	575	-	-	-	-

^1^ Sum Sq.: Sum of squares, ^2^ d.f.: degrees of freedom, ^3^ Mean Sq.: Mean square, ^4^ PCR: Percentage contribution ratio (%).

**Table 4:** Standard five-way ANOVA table results for *Rz* applying two-factor interaction model.

Analysis of Variance						
Source	Sum Sq.	d.f.	Mean Sq.	F value	Prob > F	PCR (%)
DOC	156.7400	1	156.7400	557.9044	0.0000	7.54%
*V_c_*	0.1768	1	0.1768	0.6291	0.4280	0.01%
Substrate/Coating	25.9244	2	12.9622	46.1380	0.0000	1.25%
*r*	741.2233	1	741.2233	2638.3299	0.0000	35.66%
*f*	950.0339	3	316.6780	1127.1919	0.0000	45.70%
DOC*V_c_	0.5897	1	0.5897	2.0990	0.1480	0.03%
DOC*Substrate/Coating	13.4499	2	6.7250	23.9370	0.0000	0.65%
DOC*r	0.1032	1	0.1032	0.3673	0.5447	0.00%
DOC*f	0.0195	3	0.0065	0.0232	0.9952	0.00%
V_c_*Substrate/Coating	7.6992	2	3.8496	13.7024	0.0000	0.37%
V_c_*r	6.0209	1	6.0209	21.4309	0.0000	0.29%
V_c_*f	2.5994	3	0.8665	3.0841	0.0270	0.13%
Substrate/Coating*r	148.9803	2	74.4901	265.1421	0.0000	7.17%
Substrate/Coating*f	16.6846	6	2.7808	9.8979	0.0000	0.80%
r*f	8.6256	3	2.8752	10.2341	0.0000	0.41%
Error	152.5527	543	0.2809	-		
Total	2231.4233	575				

**Table 5:** Statistics analysis to select the fitting model for *Ra.*

Source	Standard Deviation	*R*²	Adjusted *R*²	Predicted *R*²	PRESS ^1^	Model
Linear	0.2004	0.8709	0.8622	0.8493	4.17	-
2FI	0.0878	0.9791	0.9735	0.9643	0.9891	Suggested
Quadratic	0.0884	0.9791	0.9732	0.9632	1.02	Aliased

^1^ PRESS: Predicted Residual Sum of Squares for the model.

**Table 6:** Statistics analysis to select the fitting model for *Rz.*

Source	Standard Deviation	*R*²	Adjusted *R*²	Predicted *R*²	PRESS	Model
Linear	0.7775	0.8588	0.8493	0.8354	62.72	-
2FI	0.4906	0.9526	0.9400	0.9208	30.17	Suggested
Quadratic	0.4905	0.9533	0.9400	0.9198	30.55	Aliased

**Table 7:** ANOVA for reduced 2FI model of the *Ra* response.

Source	Sum of Squares	d.f.	Mean Square	*F*-Value	*p*-Value	Significance
Model	27.07	11	2.46	348.49	<0.0001	significant
A-DOC	0.2008	1	0.2008	28.42	<0.0001	-
C-*r*	7.72	1	7.72	1093.54	<0.0001	-
D-*f*	15.87	1	15.87	2246.37	<0.0001	-
E-Substrate/Coating	0.3037	2	0.1518	21.50	<0.0001	-
DOC*r	0.0546	1	0.0546	7.73	0.0067	-
r*f	0.0586	1	0.0586	8.29	0.0050	-
Substrate/Coating*r	2.80	2	1.40	198.40	<0.0001	-
Substrate/Coating*f	0.0649	2	0.0324	4.59	0.0128	-
Residual	0.5933	84	0.0071	-	-	-
Cor Total	27.67	95	-	-	-	-

The Model F-value of 348. 49 implies the model is significant. There is only a 0.01% chance that an *F*-value this large could occur due to noise.

**Table 8:** ANOVA for reduced 2FI model of the *Rz* response.

Source	Sum of Squares	d.f.	Mean Square	*F*-Value	*p*-Value	Significance
Model	362.24	15	24.15	102.36	<0.0001	significant
A-DOC	28.17	1	28.17	119.39	<0.0001	-
B-*V_c_*	0.0040	1	0.0040	0.0170	0.8967	-
C-*r*	127.84	1	127.84	541.88	<0.0001	-
D-*f*	165.67	1	165.67	702.27	<0.0001	-
E-Substrate/Coating	5.63	2	2.82	11.93	<0.0001	-
DOC*Substrate/Coating	2.05	2	1.03	4.35	0.0161	-
V_c_*Substrate/Coating	1.83	2	0.9155	3.88	0.0246	-
r*f	1.94	1	1.94	8.23	0.0053	-
Substrate/Coating*r	26.78	2	13.39	56.75	<0.0001	-
Substrate/Coating*f	2.32	2	1.16	4.92	0.0096	-
Residual	18.87	80	0.2359	-	-	-
Cor Total	381.11	95	-	-	-	-

The Model F-value of 102. 36 implies the model is significant. There is only a 0.01% chance that an *F*-value this large could occur due to noise.

**Table 9:** Goals and parameter ranges for optimization of *Ra* and *Rz* responses.

Name	Goal	Lower Limit	Upper Limit
A:*DOC*	is in range	0.5	1
B:*V_c_*	is in range	150	250
C:*r*	is in range	0.4	0.8
D:*f*	is in range	0.12	0.2
E:Substrate/Coating	is in range	WC/PVD	Cermet/PVD
*Ra*	minimize	0.6	2
*Rz*	minimize	3.7	5.5

**Table 10:** Solutions for the *Ra* response optimization.

DOC	*V_c_*	*r*	*f*	Substrate/Coating	*Ra*	Desirability
0.500	250.000	0.800	0.120	WC/PVD	0.647	0.966
0.500	229.375	0.800	0.120	WC/PVD	0.647	0.966
0.980	240.439	0.800	0.120	WC/PVD	0.689	0.936
0.995	242.386	0.800	0.120	WC/PVD	0.690	0.935
0.500	209.819	0.800	0.120	Cermet/PVD	0.722	0.913
0.500	207.158	0.800	0.120	Cermet/PVD	0.722	0.913

**Table 11:** Solutions for the *Rz* response optimization.

DOC	*V_c_*	*r*	*f*	Substrate/Coating	*Rz*	Desirability
0.500	250.000	0.800	0.120	Cermet/PVD	3.754	0.970
0.500	248.364	0.800	0.120	Cermet/PVD	3.755	0.970
0.500	247.639	0.800	0.120	Cermet/PVD	3.755	0.969
0.500	245.607	0.800	0.120	Cermet/PVD	3.756	0.969
0.500	249.999	0.800	0.120	WC/PVD	3.858	0.912
0.500	247.507	0.800	0.120	WC/PVD	3.867	0.907

**Table 12:** Goals and parameter ranges for optimization of *Ra* and *Rz* responses.

Name	Goal	Lower Limit	Upper Limit
A:DOC	is equal to 1		
B:*Vc*	is equal to 250		
C:*r*	is in range	0.4	0.8
D:*f*	is equal to 0.2		
E:Substrate/Coating	is in range	WC/PVD	Cermet/PVD
*Ra*	minimize	1.7	4
*Rz*	minimize	8.1	10

**Table 13:** Solutions of the *Ra* response optimization.

DOC	*V_c_*	*r*	*f*	Substrate/Coating	*Ra*	Desirability
1.000	*250.000*	0.800	0.200	WC/PVD	1.749	0.979
1.000	*250.000*	0.800	0.200	Cermet/PVD	1.869	0.927
1.000	*250.000*	0.800	0.200	WC/CVD	1.958	0.888
1.000	*250.000*	0.400	0.200	WC/CVD	2.186	0.789
1.000	*250.000*	0.400	0.200	Cermet/PVD	2.666	0.580
1.000	*250.000*	0.400	0.200	WC/PVD	2.794	0.525

**Table 14:** Solutions of the *Rz* response optimization.

DOC	*V_c_*	*r*	*f*	Substrate/Coating	*Rz*	Desirability
1.000	250.000	0.800	0.200	WC/PVD	8.136	0.981
1.000	250.000	0.800	0.200	Cermet/PVD	8.149	0.974
1.000	250.000	0.800	0.200	WC/CVD	8.910	0.574

## Supplementary Materials

The following are available online at https://www.mdpi.com/1996-1944/13/22/5231/s1, Figure S1: Main effects plot for surface roughness means: (a) *Ra*; (b) *Rz;* Figure S2: Graphs of the multiple comparison of the means, considering Substrate/Coating and cutting speed: (a) and (b), *Ra*; (c) and (d), *Rz.*

## Nomenclature

AISI	American Iron and Steel Institute
Al_2_O_3_	Aluminum oxide
ANOVA	Analysis of Variance
CBN	Carbon Boron Nitride
CVD	Chemical Vapor Deposition
DOE	Design of Experiments
DOC	Depth of cut (mm)
*f*	feed rate (mm/rev)
ISO	International Organization for Standardization
MT-CVD	Moderate Temperature Chemical Vapor Deposition
PVD	Physical Vapor Deposition
*r*	tool nose radius (mm)
*Ra*	Arithmetic Mean Value of Profile (µm)
*Rz*	ISO ten-point height (µm)
RSM	Response Surface Methodology
TiAlN	Titanium Aluminum Nitride
TiN	Titanium Nitride
TiCN	Titanium Carbo-Nitride
*V_c_*	cutting speed (m/min)
WC	Tungsten carbide
