# Supplementary material for: Assessment of CVD- and PVD-Coated Carbides and PVD-Coated Cermet Inserts in the Optimization of Surface Roughness in Turning of AISI 1045 Steel
Source: Materials (Basel). 2020 Nov 19;13(22):5231. doi: 10.3390/ma13225231 (PMC7699436; doi:10.3390/ma13225231)
Supplement: Supplementary file 1 [file materials-13-05231-s001.pdf]

materials

MDPI

Article

Assessment of CVD- and PVD-Coated Carbides and PVD-Coated Cermet Inserts in the Optimization of Surface Roughness in Turning of AISI 1045 Steel

Evandro Paese [1], Martin Geier [2], Fabiano R. Rodrigues [3], Tadeusz Mikolajczyk [4] and Mozammel Mia [5,*]

[1] Departamento de Engenharia Mecânica, Universidade de Caxias Sul, Campus Universitário da Região dos Vinhedos, Bento Gonçalves, Rio Grande do Sul 95705-266, Brazil; epaese@ucs.br
[2] Departamento de Engenharia Mecânica, Universidade Federal do Rio Grande do Sul, Porto Alegre, Rio Grande do Sul 90050-170, Brazil; martin.geier@ufrgs.br
[3] Programa de Pós-Graduação em Engenharia Mecânica, Universidade de Caxias Sul, Caxias do Sul, Rio Grande do Sul, Brazil; frrodri1@ucs.br
[4] Department of Production Engineering, UTP University of Science and Technology, 85-796 Bydgoszcz, Poland; tami@utp.edu.pl
[5] Department of Mechanical Engineering, Imperial College London, South Kensington, London SW7 2AZ, UK
* Correspondence: m.mia19@imperial.ac.uk

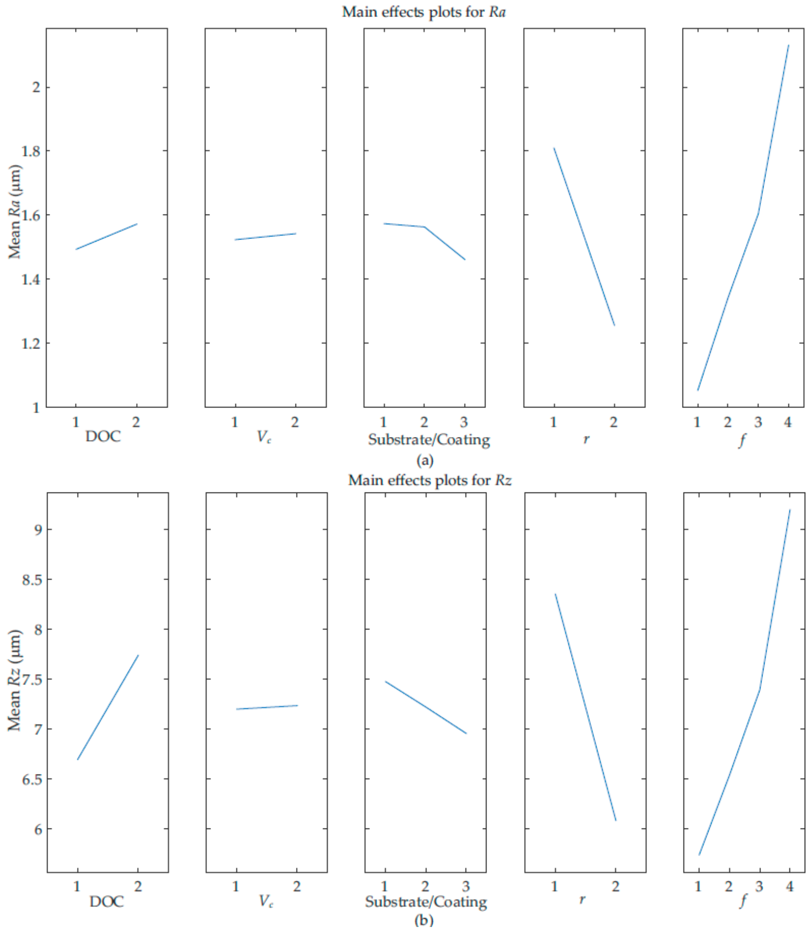

Figure S1. Main effects plot for surface roughness means: (**a**) *Ra*; (**b**) *Rz*.

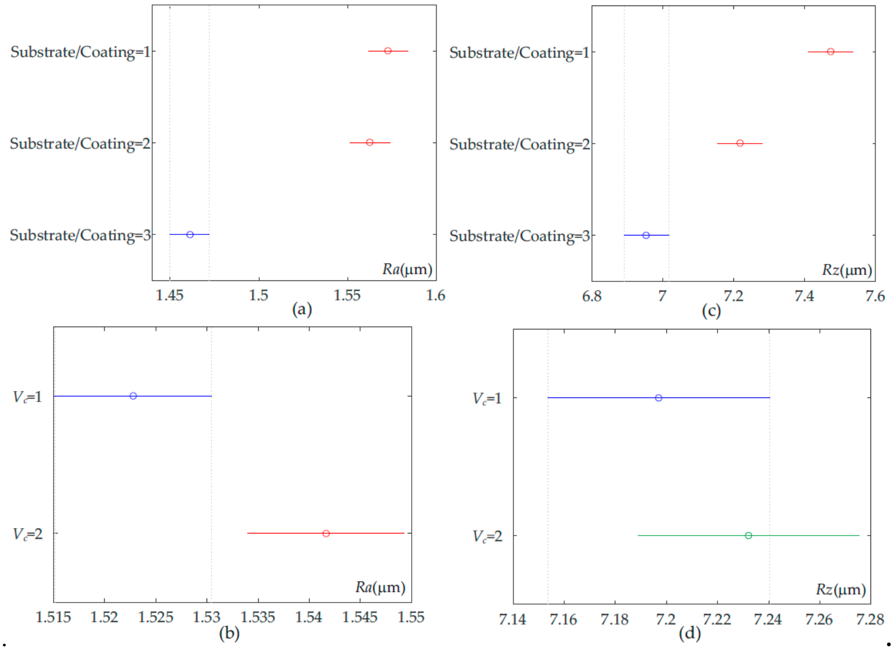

Figure S2. Graphs of the multiple comparison of the means, considering Substrate/Coating and cutting speed: (**a**) and (**b**), *Ra;* (**c**) and (**d**), *Rz.*

© 2020 by the authors. Licensee MDPI, Basel, Switzerland. This article is an open access article distributed under the terms and conditions of the Creative Commons Attribution (CC BY) license (http://creativecommons.org/licenses/by/4.0/).
